# Genome sequences of two *Bacillus anthracis* strains utilized as veterinary vaccines in China

**DOI:** 10.1128/mra.00281-24

**Published:** 2024-06-25

**Authors:** Yufei Lyu, Shuo Yu, Lingwei Zhu, Yan Guo, Shujuan Yu, Chao Pan, Li Zhu, Hengliang Wang, Dongshu Wang, Xuejun Guo, Xiankai Liu

**Affiliations:** 1 State Key Laboratory of Pathogen and Biosecurity, Beijing Institute of Biotechnology, Beijing, China; 2 Laboratory of Advanced Biotechnology, Beijing, China; 3 Key Laboratory of Jilin Province for Zoonosis Prevention and Control, Changchun Veterinary Research Institute, Chinese Academy of Agricultural Sciences, Changchun, China; The University of Arizona, Tucson, Arizona, USA

**Keywords:** *Bacillus anthracis*, veterinary vaccine strain, China, whole-genome sequencing, hybrid assembly

## Abstract

In this report, we present the complete genome sequences of two *Bacillus anthracis* strains utilized as veterinary vaccines in China. The sequencing was conducted using a hybrid assembly methodology that combined Illumina short reads and PacBio long reads. This approach provides a high-quality representative sequence for the strains mentioned above.

## ANNOUNCEMENT

In China, two veterinary vaccine strains (CVCC40202 and CVCC40205) are commonly used to protect livestock from anthrax infection. CVCC40202 was attenuated by Rentian in Japan through successive passages at elevated temperatures before its introduction to the Harbin Veterinary Research Institute in 1950 (1). CVCC40205 was introduced from the Indian Veterinary Research Institute to the China Institute of Veterinary Drug Control in 1954. Both veterinary vaccine strains have been deposited at the China Veterinary Culture Collection Center (http://cvcc.ivdc.org.cn/). In this study, whole genome sequencing of CVCC40202-BJIB02 (referred to as BJIB02) and CVCC40205-BJIB05 (referred to as BJIB05) was conducted.

One loopful of droplets from frozen spore solution was streaked on a Luria-Bertani (LB) agar plate and incubated overnight at 30°C. Then, a colony was inoculated into LB broth at 30°C (220 r/min) for 12 h for DNA extraction using the Promega Wizard Genomic DNA Purification Kit (Promega, Madison, WI, USA). NEBNext Ultra II DNA Library Prep Kit (NEB, USA) was used to construct the Illumina sequencing library. After the gDNA sample passed the test, it was broken into 10 KB fragments using a g-Tube and purified with 0.45× magnetic beads. The purified samples were used to prepare a PacBio sequencing library using SMRTbell Express Template Prep Kit 2.0 (PacBio, USA). Whole-genome sequencing was performed using a hybrid assembly approach of Pacific Biosciences (PacBio) Sequel II and Illumina NovaSeq 6000. The short-reads from the Illumina platform were quality-filtered by SOAPnuke (v2.1.0) (2). The subreads from the PacBio platform were processed using the CCS model of SMRTlink (v11.0.0). The PacBio data were assembled using flye (v2.9) (3) and then the assembly was polished with Illumina short reads using Pilon (v1.24) (4). The genome sequence was annotated with the National Center for Biotechnology Information Prokaryotic Genome Annotation Pipeline (v6.6). Additional data about the two genomes are listed in Table 1.

**TABLE 1:** Genome characteristics of two Chinese anthrax veterinary vaccine strains

Features	CVCC40202-BJIB02	CVCC40205-BJIB05	CVCC40202-BJIB02			CVCC40205-BJIB05	
			Chromosome	pXO1	pXO2	Chromosome	pXO1
Illumina NovaSeq 6000							
Total no. of reads	7,804,200	8,067,218					
Avg read length (bp)	150	150					
SRA accession no.	SRR28019344	SRR28037805					
BioSample accession no.	SAMN39999256	SAMN39999450					
BioProject accession no.	PRJNA1078397	PRJNA1078406					
							
PacBio Sequel II							
Total no. of reads	664,005	741,811					
Avg read length (bp)	9,290	8,981					
Read N50 (bp)	9,413	9,014					
SRA accession no.	SRR28019343	SRR28037804					
BioSample accession no.	SAMN39999256	SAMN39999450					
BioProject accession no.	PRJNA1078397	PRJNA1078406					
							
Assembly genome							
Total length (bp)			5,228,927	181,710	94,722	5,226,417	181,633
Coverage (X)			46.21	72.12	23.38	45.61	99.04
GC content (%)			35.38	32.53	33.05	35.38	32.53
GenBank accession no.			CP145726	CP145727	CP145728	CP145729	CP145730

**Fig 1:**
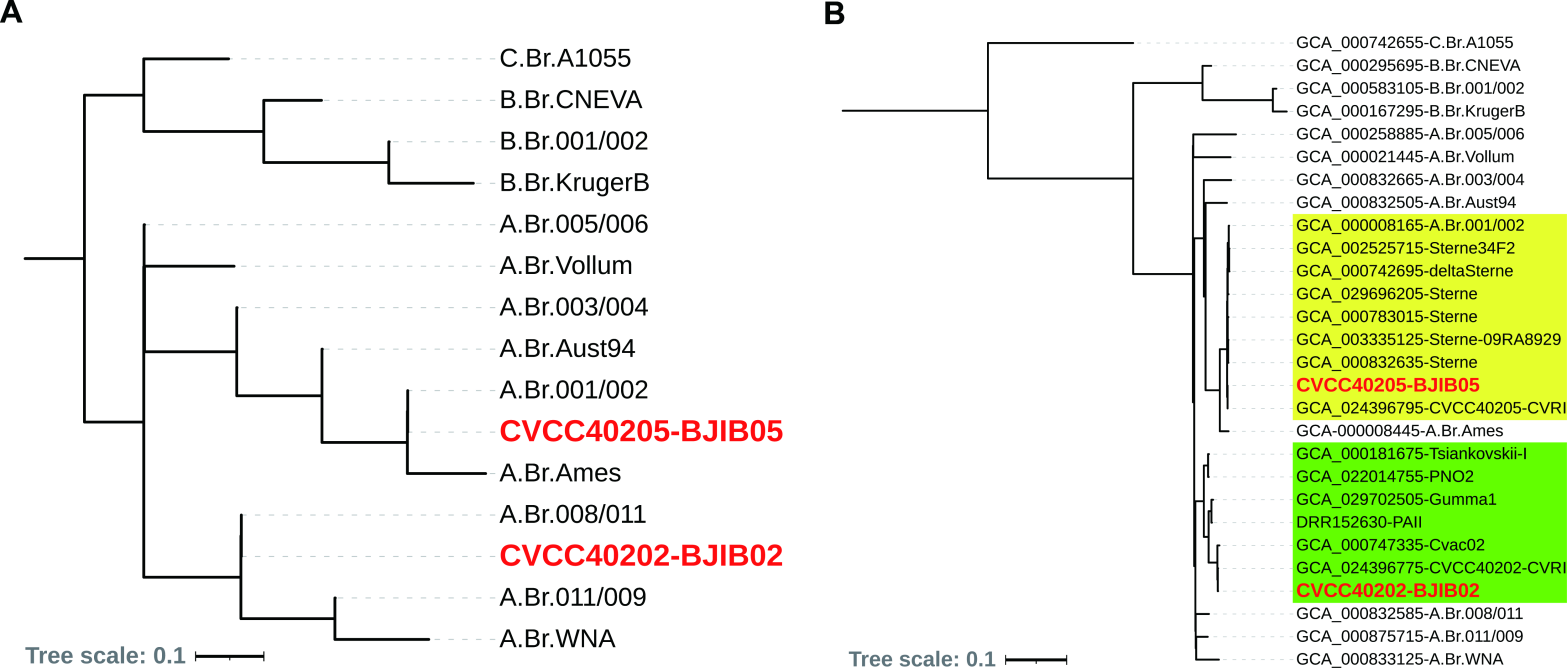
Phylogenetic relationships of strains CVCC40202-BJIB02 and CVCC40205-BJIB05. (**A**) The relationship between the 13 canonical SNP subgroups and the two Chinese veterinary *B. anthracis* vaccine strains. (**B**) Phylogenetic analysis based on genome-wide SNPs. The clades that CVCC40202-BJIB02 and CVCC40205-BJIB05 belong to were highlighted in green and yellow, respectively.

*In silico* canonical single-nucleotide polymorphism (canSNP) and genome-wide SNP analysis were performed on the two strains and other selected *Bacillus anthracis* strains (5, 6). The results indicated that BJIB02 belonged to A.Br.011/009 lineage, while BJIB05 belonged to A.Br.001/002 subgroup (Fig. 1A).

For whole-genome SNP analysis, the Harvest Suite (V1.1.2) was used to create a variant calling file (7) and MEGA was used to align the concatenated SNP sequences (8). FastTree (v2.1.10) was used to construct a maximum-likelihood tree (9), which was further edited using online iTOL tools (https://itol.embl.de). The results indicate that BJIB02 is very close to GCA_024396775-CVCC40202-CVRI and GCA_000747335-Cvac02, all of which belong to the No. II Chinese veterinary anthrax vaccine strain (10). The primary distinctions among the three genomes are the sequencing methods and the laboratory of origin. These three strains, along with five other strains including two Japanese strains of PAII and Gumma1 (11), one Russian strain Tsiankovskii-I, and one Chinese strain PNO2 (12), are clustered into a large clade. BJIB05 is very close to GCA_024396775-CVCC40205-CVRI and they clustered into a large clade which includes many Sterne strains (Fig. 1B). Default parameters were used for all software unless otherwise specified.

## Data Availability

The genomic sequences are available in NCBI GenBank under BioProject accession numbers PRJNA1078397 and PRJNA1078406. The raw reads for Illumina NovaSeq and PacBio have been deposited in the SRA database under accession numbers SRR28019344 and SRR28019343 for BJIB02, respectively; and SRR28037805 and SRR28037804 for BJIB05, respectively.
